# Expectations, effect and experiences of an easily accessible self-management intervention for people with chronic pain: study protocol for a randomised controlled trial with embedded qualitative study

**DOI:** 10.1186/s13063-016-1462-6

**Published:** 2016-07-18

**Authors:** Torunn Hatlen Nøst, Aslak Steinsbekk, Ola Bratås, Kjersti Grønning

**Affiliations:** Centre for Health Promotion Research, Norwegian University of Science and Technology, 7491 Trondheim, Norway; Department of Nursing Science, Norwegian University of Science and Technology, 7491 Trondheim, Norway; Department of Public Health and General Practice, Norwegian University of Science and Technology, 7491 Trondheim, Norway

**Keywords:** Chronic pain, Self-management, Patient activation, Health promotion, Primary care, Health services accessibility, Easy access, (MeSH): chronic pain, Self-care, Patient participation, Health promotion, Primary health care, Health services accessibility, Health-care quality, Access and evaluation

## Abstract

**Background:**

People struggling with chronic pain may benefit from different types of non-pharmacological interventions such as self-management courses. Self-management courses aim to increase participants’ skills and knowledge in managing chronic conditions. Community health-care services in Norway have increasingly established Healthy Life Centres (HLCs) to offer easily accessible interventions to people in need of support to better handle a life with chronic illness. The aim of this trial is to investigate the expectations, effect and experience of an easily accessible, group-based self-management course delivered at a HLC for people with chronic pain.

**Methods/Design:**

This is an open pragmatic two-armed randomised controlled trial with an embedded qualitative study. The intervention is a self-management course comprising education, discussions, exchange of experiences between the participants, and physical movement exercises. The control group is offered a drop-in outdoor physical activity. The intervention period is 6 weeks. The primary outcome is patient activation measured by the patient activation measure (PAM). The secondary outcomes include measures of self-efficacy, pain and quality of life. Data will be collected at baseline, and after 3, 6 and 12 months. Using a mixed linear model, the number needed in each arm to achieve a power of 80 % becomes 55. To allow for dropout, the aim is to include 120 participants. Analysis will be done using mixed linear models. In the embedded qualitative study, we will perform semi-structured face-to-face interviews with a sample from both trial arms before randomisation and after 3 and 12 months. The topics elaborated will be motivation for participation and experiences with the activity related to possible changes in managing and coping with chronic pain.

**Discussion:**

There is need for more knowledge on interventions delivering self-care support in an easily accessible way that aim to reach those in need of this kind of health service. This trial will produce important knowledge on the effect and the experiences of participants in such an easily accessible self-management course delivered in Norwegian public primary care.

**Trial registration:**

ClinicalTrials.gov: NCT02531282. Registered on 21 August 2015.

**Electronic supplementary material:**

The online version of this article (doi:10.1186/s13063-016-1462-6) contains supplementary material, which is available to authorized users.

## Background

The rising prevalence in long-term conditions (LTCs) presents a major challenge in society and health-care services worldwide [1, 2]. This has led to increased attention towards interventions supporting self-care as effective approaches and core components in the health-care service [3, 4].

Self-care indicates the actions people take independently to lead a healthy lifestyle and how they engage in behaviours that affect their health [3, 5, 6]. To take on the responsibility that lies within self-care, people need knowledge and skills on how to manage their own health, highlighting the importance of being active and engaged as patients [6, 7]. This can be seen as an ideological shift from patients as passive recipients of treatment to patients being empowered individuals managing their own health [8]. Although people manage their health mostly outside the health-care services, people with LTCs typically need ongoing treatment over decades. They may therefore benefit at varying times from interventions supporting self-care [6, 7].

This benefit has led to an increase in interventions to support self-care, typically called self-care support or self-management interventions [6, 9]. In the following, we will use the term self-management interventions when referring to these activities. Self-management interventions are offered in various forms. They can be led by lay persons or professionals, be generic or disease specific, and be delivered in groups or to individuals [5, 9]. They have been shown to result in improvements in various domains such as in the participants’ engagement, self-efficacy, mood, physical symptoms and function, and reduced health-service utilisation [4, 7, 10].

However, reaching and engaging those likely to benefit from participation in self-management interventions have been described as insufficient, leading to high attrition rates and low uptake [11, 12]. Difficulties in accessing health services and health-care personnel are described by patients as barriers to participation in different types of self-management interventions [13–15]. As self-care support is acknowledged as a key in management of LTCs, a sustainable number of resources has been invested in offering these interventions [6, 16]. Delivering easily accessible self-management interventions, therefore, seems to be significant in providing a sustainable health service, and primary care and community health-care services seem to be the suitable arenas for them [12].

In a Norwegian context, group-based patient education as a self-management activity has traditionally been offered in hospitals (see general introduction in [17]). Recent health reforms promote support for self-care to be carried out also in the local communities [18]. This action aims to offer interventions that are easy to access. In addition, the Norwegian Directorate of Health has since 2004 encouraged municipalities to establish Healthy Life Centres (HLCs) as part of their public health care [19]. HLCs are low-threshold health-care services delivering easily accessible activities and interventions in the communities. These services aim to support people at risk with health behavioural changes and in managing chronic conditions [19, 20]. The government writes that the theoretical framework of HLCs’ interventions should be in salutogenesis [20], where strengthening people’s capacities to use their own and available health resources is central [21].

One group utilising HLCs comprises people with long-lasting pain. Long-lasting or chronic pain is a widespread LTC that affects up to one-third of the population [22–24]. The condition affects the physical, social and psychological dimensions in life [25–27]. Pain sufferers often describe problems of sleeplessness, depression, poor quality of life, and exhaustion, as well as interference with physical ability, social relations and work life [23, 28, 29]. An increase in the number of people struggling with chronic pain is documented [22, 23]. It has an impact on society in terms of increased health-care utilisation, sick leave and early retirement [1, 23, 30].

As chronic pain has an impact on many aspects in a person’s life, different treatment approaches are needed. Current recommendations focus on both pharmacological and non-pharmacological interventions [27, 31]. One central approach in non-pharmacological interventions is support for self-management, aiming to increase the individual’s ability to manage his or her pain in everyday life. Because today’s treatments provide modest improvement in pain and minimum improvements in physical and emotional functioning [27, 32], a substantial number of people are left to struggle with chronic pain in everyday life. This difficulty pinpoints the importance of communities addressing the need for health care for this group of people, preferably by delivering easily accessible interventions, like the ones offered in the Norwegian HLCs. However, to our knowledge, nothing has been published on the effect of and on participant experiences with self-management interventions addressing chronic pain that are delivered in a HLC.

### Aims and objectives

This trial’s overall aim is to investigate the expectations, effect and experiences of an easily accessible self-management course for people with chronic pain delivered at a HLC in community public health care.

The primary objective is to study in a randomised controlled trial (RCT) the hypothesis that an easily accessible group-based self-management course for people with chronic pain is more effective at patient activation than a drop-in group-based easy outdoor physical activity.

The embedded qualitative study seeks to investigate the expectations for participation in a self-management course in this setting. In addition, we will investigate the participants’ experiences with the intervention and changes in how they manage their lives with chronic pain.

## Methods/Design

**Fig. 1:**
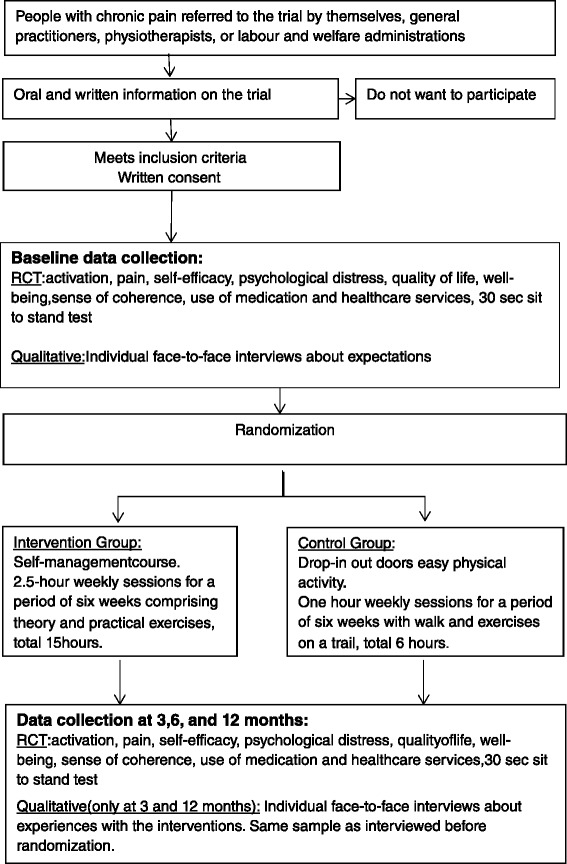
Flow chart of the trial

This is an open pragmatic parallel two-arm RCT with an embedded qualitative study (Fig 1). The intervention is complex, as it comprises multiple interacting components [33, 34]. The Medical Research Council guidelines for complex interventions [34] and the SPIRIT guideline (Standard Protocol Items: Recommendations for Interventional Trials) with its checklist [35] were consulted when writing the protocol.

### Setting

HLCs (“Frisklivssentral” in Norwegian) are a public service offered by municipalities as part of the Norwegian community health-care system [19]. The HLCs strive to offer easily accessible interventions with few barriers for participation. They aim to give a low-threshold health-care service that people can attend with or without referral from others. In addition, a HLC receives referrals from general practitioners, physiotherapists and the Norwegian Labour and Welfare Administration [20].

Our project is situated at a HLC in a city in central Norway serving a population of approximately 185,000 inhabitants. The centre has 5.5 positions occupied by multidisciplinary health professionals with a bachelor’s or master’s degree. The HLC offers several group-based activities and interventions, e.g. indoor and outdoor physical activity, healthy diet courses, smoking cessation programmes and courses focusing on coping with depression or anhedonia. The drop-in physical activity offered to the control group is an example of an activity at the HLC where people can attend without referral or further commitment. In cooperation with patient organisations, the HLC staff developed the self-management course constituting the intervention arm in this trial.

### Inclusion criteria

The inclusion criteria are adults of 18 years or older who can self-report challenges with pain for more than 3 months and are able to take part in group discussions in Norwegian. The 3-month criterion is set according to the International Association for the Study of Pain definition of chronic pain as being ‘pain without apparent biological value that has persisted beyond the normal tissue healing time over 3 months’ [36–38]. To enhance external validity, the inclusion criteria are simple and broad, as in similar pragmatic trials [32, 38] and because they mirror practice at the HLC.

### Exclusion criteria

The exclusion criteria are not being able to take part in the activity offered to the control group (1 hour of easy physical activity, e.g. walking), chronic pain arising from malignant diseases, and not having the capacity to consent and participate.

### Recruitment

Information on the opportunity to refer people to the trial will be given to physiotherapists, general practitioners, Norwegian Labour and Welfare Administration and other important organisations in the municipality. To promote the possibility of self-referral, flyers and posters with information on the trial will also be distributed to offices and waiting areas of general practitioners and physiotherapists, and to Norwegian Labour and Welfare Administration offices. Advertisements will be placed in newspapers, on social media and websites, as well as in emails to relevant patient organisations.

The first author will be responsible for checking the participants’ eligibility criteria before enrolment. Participants will be enrolled until the target sample size is reached.

Recruitment to RCTs is acknowledged as challenging [10]. To make participation in the trial more appealing, all participants are offered an activity. Amounts normally paid for attending interventions in the HLC (normally about $34/€31) will be covered by the trial. No other financial support is planned.

### Interventions

#### Self-management course

The content of the self-management course includes theoretical education, group discussions based on the theoretical input, sharing of experiences and movement exercises. The staff developed the course based on recommendations found in the literature (e.g. [39–41]) and added elements from cognitive behavioural therapy focusing on the participants’ thoughts, emotions and actions. The theoretical part of the course comprises pain theory, barriers in everyday life due to chronic pain, problem-solving, goal-setting and techniques to deal with fatigue, poor sleep, frustrations and isolation. The movement exercises in each session aim to improve balance, posture and breathing, providing the participants with techniques to increase body awareness and the ability to relax. These techniques are essential in Norwegian psychomotor physiotherapy [42] on which this element of the course is built.

Guidelines for how to carry out the course have been developed to help the instructors, ensuring all groups are offered the same content and material (see Additional file 1).

The course is delivered as 2.5-hour weekly group workshops during the daytime for a period of 6 weeks, reaching a total of 15 hours, with 8–10 participants in each group. Two employees who have been dedicated to facilitating the course have a professional background as physiotherapists. All workshops start with the theoretical elements followed by the part of the course introducing movement exercises in a training room. Between the parts, there will be breaks for social interaction. Between each workshop, the participants will be given homework, e.g. working on their goal-setting plan.

#### Control group

Participants in the control group will be offered a drop-in outdoor easy physical activity, comprising walking and simple strength exercises. The activity will be adjusted to the participants’ physical ability, to keep it both easily accessible and rewarding. The rationale for choosing this activity is that the HLC already offers this activity as a drop-in service without registration or further commitment from people. Offering an activity to all participants in the trial is recognised as ethical and it is good clinical practice [43, 44]. Participation in the control group activity is voluntary, in line with the drop-in policy at the HLC.

The easy physical activity will be delivered as a weekly 1-hour session for a period of 6 weeks. The activity will be in groups with a group size similar to the intervention (8–10 participants in each group) and led by two instructors with skills in physical activity. The group will meet outdoors on a trail popular for walking among the municipality’s inhabitants. However, due to changing weather conditions and according to conditions on the trail, the location might be changed to another area during winter.

### Documentation of the delivery of the intervention

To document the delivery of the interventions, the instructors will complete an evaluation form for each group. They will be asked to report their experiences with the delivery, the group dynamics and whether there were any adverse events. The instructors will also report the number of participants attending each session.

### Randomisation and allocation

Randomisation to the intervention or control group will be done using a computerised Internet-based randomisation service. Previous studies on interventions in primary care indicate that more women than men participate in self-management interventions [45]. Thus, the randomisation will stratify on gender allocating men equally to both trial arms.

The first author will inform the participants of the result of the allocation by telephone or email immediately after the randomisation. After allocation to one of the trial arms, the participants are informed that there will be no possibility of changing arms.

### Ethics

The researchers have obtained approval from the director for health and social affairs in the municipality and from the Regional Committee for Medical and Health Research Ethics (2015/1030/REK sørøst). The trial will be carried out in accordance with the Helsinki Declaration [46]. The participants will be informed both orally and in writing, and written consent to participate will be collected before enrolment by the first author.

People suffering from chronic pain can be vulnerable. It will be important to assure the participants that the study will not inflict harm or include invasive interventions. Nevertheless, there is a possibility that reactions from earlier experiences can occur. The participants will, therefore, receive information on who to contact at the HLC if they need to talk to someone about their reactions.

Adverse events occurring in the sessions will be reported and registered by the instructors. Minor adverse events, e.g. participants being tearful or distressed during the activity [32], will be registered and acted upon after the group-based activity. If more serious adverse events occur, e.g. extreme distress or expressed suicidal thoughts [32], they will immediately be reported to the HLC management and followed up.

### Methodology for the RCT

#### Data collection

Data will be collected through self-report questionnaires at baseline (*t*_0_), and after 3 (*t*_1_), 6 (*t*_2_) and 12 months (*t*_3_). At baseline, the first author will be available for questions when the participants fill in the questionnaire. The first author will also supervise the physical ability test at baseline. Background variables such as age, gender, marital status, employment status, pain duration, referring institution, other diseases coded with the International Classification of Primary Care 2 (ICPC-2) [47], current medication and health-care utilisation will also be collected at baseline. At the three follow-up points (3, 6 and 12 months), the participants will be sent the questionnaires by mail. The participants will bring the completed self-administrated questionnaires to the follow-up visits, where a research assistant will supervise the physical ability test. There will be one postal reminder for each follow-up data collection point, and non-responders will be contacted by phone or email allowing a delay of up to 4 weeks.

#### Blinding

After allocation, blinding is not possible due to the intervention’s nature. However, a research assistant blinded to allocation will conduct the physical ability test at *t*_1_, *t*_2_ and *t*_3_. The research assistant will have a protocol describing how to perform the physical ability test and which data to collect (changes in marital status, employment status, and current use of health care and medication), and the participants will be asked not to divulge their allocation.

#### Outcomes

##### Primary outcome

We hypothesise that participating in the self-management course will strengthen the participants’ engagement and their knowledge of health resources, and will consequently lead to a higher level of patient activation. Therefore, the primary outcome is patient activation assessed with the patient activation measure (PAM) [48].

PAM contains 13 items representing statements to which the participants indicate their level of agreement on a four-point scale from strongly disagree to strongly agree [48]. The responses give a raw score from 13 to 52, which is calibrated to a total score between 0 and 100 using the transformation tables provided by Insignia Health [49]. A high score indicates that the participants are more activated to adopt and maintain healthy behaviours and self-management of their illness, even under stress [50].

Several studies show that PAM is useful for assessing patient engagement in the management of chronic illness and it has been shown to be sensitive to change in several groups and populations [51–54]. PAM has been translated and validated for use in a Norwegian context [55].

##### Secondary outcomes

Furthermore, we hypothesise that participating in the self-management course may influence several health dimensions leading to the following secondary outcomes:

- *Pain*: The Brief Pain Inventory [56] will be used for assessing pain. This instrument has four questions related to pain severity and seven questions assessing pain interference [56, 57]. The instrument is widely used for measuring both malignant and non-malignant pain [58, 59] and has been translated and validated for use in a Norwegian context [60–62].
- *Pain*: The overall experience of pain during the last week will be assessed with a one-item 100-mm visual analogue scale (VAS). The scale’s anchoring points are no pain (0) to intolerable pain (100) [63]. The VAS scale is widely used in the assessment of various conditions and has been validated and found reliable in the assessment of chronic pain [63].
- *Pain-related self*-*efficacy*: Self-efficacy refers to the person’s self-conception of confidence to complete activities, recognised as a central aspect in the self-management of pain [64]. We will use the Pain Self-Efficacy Questionnaire [64, 65], which has recently been translated into Norwegian [66]. It is a ten-item instrument that ascertains the individual’s level of confidence to live a normal life despite pain. Each question is scored on a scale from 0 to 6 giving a total range of 0–60, with a higher score indicating higher pain self-efficacy [64]. The scale has shown strong psychometric qualities [64, 67].
- *Anxiety and depression*: The self-rating instrument Hospital Anxiety and Depression Scale (HADS) will be used to assess anxiety and depression [68]. HADS consists of 14 items divided into two subscales, for anxiety and for depression, respectively. Each item is rated from not experiencing a symptom (0) to experiencing a symptom nearly all the time (3), giving a total score range for each subscale from 0 (best) to 21 (worst). Higher scores indicate more severe depression or anxiety [68]. HADS has been translated into Norwegian and has been found to be valid and reliable for use in a Norwegian context [69, 70]. The instrument has shown good validity and reliability for patients with musculoskeletal pain [71].
- *Quality of life*: We will use the EuroQoL (EQ-5D-5 L) to assess health-related quality of life, comprising the five dimensions of mobility, self-care, usual activities, pain/discomfort and anxiety/depression. In this trial, we will use the new version of the questionnaire, which provides five levels for answering each dimension: no problems, slight problems, moderate problems, severe problems and extreme problems. The scores provide a value based on a population tariff where 0 equates to dead and 1 equates to full health [72, 73]. The instrument has been validated in similar populations [74, 75] and in a Norwegian context [76].
- *Well-being*: To measure the overall experience of well-being, the Arizona Integrative Outcome Scale (AIOS) will be used [77]. This is a one-item 100-mm-long VAS and it is followed by an instruction to reflect on the sense of well-being during the last month. It has been translated into Norwegian for use in a similar population [54]. The scale’s anchoring points are ‘worst you have ever been’ (0) and ‘best you have ever been’ (100). AIOS has been found to be a valid measure for assessing well-being [77].
- *Sense of coherence*: To measure the participants’ sense of coherence, we will use the SOC-13 questionnaire. SOC-13 consists of 13 items with seven options for answers. The scoring ranges from 1 to 7, with a total score ranging from 13 to 91. A higher score indicates a stronger sense of coherence. The questionnaire has been translated and validated for a Norwegian context [78].
- *Physical ability*: As an objective measure of physical ability, we will use the 30-second chair to stand test. The test has been developed and validated for older adults [79] but has also been used and validated for wider populations [80–82].

#### Sample size

The translation of PAM into different languages has provided mean values for PAM in different populations [55, 83–85]. However, there is no common cut-off level for a clinically relevant change in PAM [86], and improvements in a PAM score after an intervention vary from 3 to 8 points [standard deviation (SD) 12–17] in different studies [54, 87–89]. Using a mixed linear model assuming a correlation within participants of 0.5, a difference between the groups at 12 months of 6 points, a SD of 13 [54], and a power of 80 %, the number needed in each arm becomes 55. To allow for dropout, the aim is to include 120 participants.

#### Statistical analysis

Outcome analyses will be conducted according to an intention-to-treat principle with mixed linear models. A mixed model accounts for repeated measures and potentially uses more of the data compared with an analysis of covariance. To account for within-subject correlations, participant ID will be specified as a random effect. The effect of intervention and time will be specified as a fixed effect with the levels ‘baseline’, ‘active 3 months’, ‘control 3 months’, ‘active 6 months’, ‘control 6 months’, ‘active 12 months’ and ‘control 12 months’.

In addition, a per-protocol analysis will be conducted, including participants who have been present at a minimum of three group sessions.

The confidence level is set to 95 %, and *p* ≤ 0.05 will be considered statistically significant. We will use Stata 14 [90], IBM SPSS 23 [91] and R version 2.13.1 [92] to analyse the data. A statistician blinded to group allocation will supervise the analysis.

### Methodology for the embedded qualitative study

#### Participants

A strategic sample of up to 30 of the participants enrolled in the RCT will be asked to participate in the qualitative study. The strategic sampling aims to include participants of both genders, with different durations of chronic pain and with different experiences from health-care services. The selected participants will be asked before randomisation to participate in interviews at baseline, and at the 3- and 12-month follow-ups.

#### Data collection

The semi-structured face-to-face interviews will use an interview guide with questions focusing on expectations and experiences of the intervention.

The interviews at baseline aim to investigate the participants’ expectations of participating in an intervention in this particular setting, and if relevant, in comparison to other services they have received. We will also seek to capture how they cope with chronic pain in their everyday life and ask what they characterise as good situations despite the pain and whether they have any personal goals for participating.

The interviews after 3 and 12 months will be with the same participants as in the baseline interviews. They aim to explore the participants’ experiences with the interventions and changes in their lives after participation. The participants will be asked how they experienced the allocated activity, if they found it useful, and what components were useful and why. In addition, we will ask how participants experienced this service compared to other health services they have received. There will be a focus on how they cope with chronic pain at this stage and if they have experienced changes in their knowledge, use of health resources, and their coping abilities and mindset.

#### Analysis

The interviews will be audiotaped and transcribed verbatim. The transcripts will be analysed according to systematic text condensation [93]. The analysis procedure implies decontextualisation, coding, synthesis, (condensation) and recontextualisation [93]. The analysis process will involve a research group in which a consensus on the findings aims to enhance the reliability of the findings.

## Discussion

In summary, the trial’s design with the use of a qualitative approach alongside the RCT will enable us to address the knowledge gap, from multiple viewpoints, regarding easily accessible self-management interventions for people living with chronic pain.

One argument for courses offered at the HLCs in Norway is that they should be easily accessible. In this way, those in need of support for habit changes to achieve a healthy lifestyle, or to manage a chronic condition, can be reached [20]. Thus, to mirror the current practice and reach high external validity, the trial aims to recruit participants from the people targeted by the HLCs. However, this might be a challenge due to the need to include enough participants in the RCT to reach the required sample size within the trial’s timeframe. As seen in other studies, recruiting strategies may lead to different participants from those attending existing services [94]. Also, high attrition rates and a low uptake of self-management interventions have been seen in other studies [6]. Our trial has a broad recruitment strategy that might attract participants beyond those who would normally attend activities at the HLC. Nevertheless, the recruited group will have to fulfil the inclusion criterion of chronic pain and, thus, belong to the group of people who could benefit from engaging in their own health care, which is emphasised as society is seeing a rise in LTCs [5, 7, 10].

The intervention is developed from recommendations on self-management and chronic pain [39–41], together with the HLCs’ experiences from working with people in need of support to manage health issues and with people experiencing chronic pain in everyday life. Thus, the aim is to deliver an intervention that meets the needs of the target population and increases the participants’ ability to self-manage. The embedded qualitative approach will be used to explore how the participants experienced the different elements of the intervention. Together with the information from the questionnaires in the RCT, the qualitative study will make it possible to investigate if the intentions for the intervention are met and if there are any elements that ought to be changed.

The effect of the intervention will be measured in accordance with its aim and content. The discrepancy between those emphasising self-management as key in managing LTCs and those demonstrating poor evidence of effectiveness for these interventions has been addressed with the choice of outcome measures [95]. Because the main objective for the self-management course is to provide the participants with knowledge and skills to live active and good lives despite their chronic pain, patient activation is chosen as the primary outcome. The secondary outcomes are chosen based on recommendations from the Initiative on Methods, Measurement, and Pain Assessment in Clinical Trials (IMMPACT) [57, 96], outcomes used in the evaluation of self-management interventions [97], and outcomes assessing health promotion, which is the HLC’s theoretical framework. Thus, in line with recommendations for investigating complex interventions [34], these outcomes and the knowledge from the embedded qualitative study cover a range of dimensions relating to people’s health. They include social and behavioural processes that are difficult to explore or capture using quantitative methods alone [98].

In conclusion, this trial is a comprehensive investigation into interventions that are recommended to meet the challenges and demands posed by the increasing number of people with long-term chronic conditions.

## Trial status

Enrolment for the trial began in August 2015 and recruitment is still in progress. Data collection will continue until approximately December 2017.

## Abbreviations

AIOS, Arizona Integrative Outcome Scale; HADS, Hospital Anxiety and Depression Scale; HLC, Healthy Life Centre; LTC, long-term condition; PAM, patient activation measure; RCT, randomised controlled trial; SD, standard deviation; VAS, visual analogue scale

## Additional file

Additional file 1: Guidelines for the self-management course. Description and support for instructors on how to deliver the self-management course. (DOCX 20 kb)

## References

[1] World Health Organization. Global status report on noncommunicable diseases 2010: description of the global burden of NCDs, their risk factors and determinants. Geneva: World Health Organization; 2011. http://www.who.int/nmh/publications/ncd_report2010/en. Accessed 15 Feb 2016.

[2] World health statistics 2012. Geneva: World Health Organization; 2012.

[3] Kennedy A, Rogers A, Bower P (2007). Support for self care for patients with chronic disease. BMJ.

[4] Kennedy A, Reeves D, Bower P, Lee V, Middleton E, Richardson G (2007). The effectiveness and cost effectiveness of a national lay-led self care support programme for patients with long-term conditions: a pragmatic randomised controlled trial. J Epidemiol Community Health.

[5] Barlow J, Wright C, Sheasby J, Turner A, Hainsworth J. Self-management approaches for people with chronic conditions: a review. Patient Educ Couns. 2002;48:177–87. 10.1016/s0738-3991(02)00032-0 12401421

[6] Boger E, Ellis J, Latter S, Foster C, Kennedy A, Jones F (2015). Self-management and self-management support outcomes: a systematic review and mixed research synthesis of stakeholder views. PLoS One.

[7] Newman S, Steed L, Mulligan K (2004). Self-management interventions for chronic illness. Lancet.

[8] Dwaarswaard J, Bakker EJM, van Staa AL, Boeije HR. Self-management support from the perspective of patients with a chronic condition: a thematic synthesis of qualitative studies. Health Expect. 2015;2:194-208. doi:10.1111/hex.12346. 10.1111/hex.12346 PMC5055271 25619975

[9] De Silva D (2011). Evidence: helping people help themselves. A review of the evidence considering whether it is worthwhile to support self-management.

[10] Carnes D, Homer KE, Miles CL, Pincus T, Underwood M, Rahman A, Taylor SJC. Effective delivery styles and content for self-management interventions for chronic musculoskeletal pain. A systematic review. Clin J Pain. 2012;28:344–54. 10.1097/AJP.0b013e31822ed2f3 22001667

[11] Schulman-Green D, Jaser SS, Park C, Whittemore R. A metasynthesis of factors affecting self-management of chronic illness. J Adv Nurs. 2016;7:1469-89. doi:10.1111/jan.12902. 10.1111/jan.12902 PMC4891247 26781649

[12] Kennedy A, Bower P, Reeves D, Blakeman T, Bowen R, Chew-Graham C (2013). Implementation of self-management support for long term conditions in routine primary care settings: cluster randomised controlled trial. BMJ.

[13] Lalonde L, Choinière M, Martin E, Lévesque L, Hudon É, Bélanger D (2015). Priority interventions to improve the management of chronic non-cancer pain in primary care: a participatory research of the ACCORD program. J Pain Res.

[14] Liddy C, Blazkho V, Mill K (2014). Challenges of self-management when living with multiple chronic conditions. Can Fam Physician.

[15] Jerant A, von Friederichs-Fitzwater MM, Moore M (2005). Patients’ perceived barriers to active self-management of chronic conditions. Patient Educ Couns.

[16] Panagioti M, Richardson G, Small N, Murray E, Rogers A, Kennedy A, Newman S, Bower P. Self-management support interventions to reduce health care utilisation without comprising outcomes: a systematic review and meta-analysis. BMC Health Serv Res. 2014;14:356. 10.1186/1472-6963-14-356 PMC4177163 25164529

[17] Bossy D, Knutsen IR, Rogers A, Foss C. Group affiliation in self-management: support or threat to identity? Health Expect. 2016. doi:10.1111/hex.12448. 10.1111/hex.12448 PMC5217888 26868829

[18] The Norwegian Ministry of Health and Care Services (2015). The primary health and care services for tomorrow – localised and integrated. Minist Health Care Serv.

[19] Denison E, Underland V, Berg RC, Vist GE (2014). Effects of more than three months organized follow-up on physical activity and diet for people with increased risk of lifestyle related disease. Report from Kunnskapssenteret.

[20] The Norwegian Directorate of Health. Guidelines for municipal Healthy Life Centers. The Ministry of Health and Care Services; 2013. https://helsedirektoratet.no/Lists/Publikasjoner/Attachments/53/IS-1896-Frisklivsveileder.pdf. Accessed 15 Mar 2016.

[21] Lindström B, Eriksson M (2005). Salutogenesis. J Epidemiol Community Health.

[22] Breivik H, Collett B, Ventafridda V, Cohen R, Gallacher D (2006). Survey of chronic pain in Europe: prevalence, impact on daily life and treatment. Eur J Pain.

[23] O’Brien T, Breivik H. The impact of chronic pain – European patients’ perspective over 12 months. Scand J Pain. 2012;3:23–9. 10.1016/j.sjpain.2011.11.004 29913762

[24] Reid KJ, Harker J, Bala MM, Truyers C, Kellen E, Bekkering GE, Kleijnen J. Epidemiology of chronic non-cancer pain in Europe: narrative review of prevalence, pain treatments and pain impact. Curr Med Res Opin. 2011;27:449–62. doi:10.1185/03007995.2010.545813. 10.1185/03007995.2010.545813 21194394

[25] Dysvik E, Kvaløy JT, Furnes B (2013). A mixed-method study exploring suffering and alleviation in participants attending a chronic pain management programme. J Clin Nurs.

[26] Breivik H, Eisenberg E, O’Brien T (2013). The individual and societal burden of chronic pain in Europe: the case for strategic prioritization and action to improve knowledge and availability of appropriate care. BMC Public Health.

[27] Turk DC, Wilson HD, Cahana A (2011). Treatment of chronic non-cancer pain. Lancet.

[28] Debono DJ, Hoeksema LJ, Hobbs RD (2013). Caring for patients with chronic pain: pearls and pitfalls. J Am Osteopath Assoc.

[29] Fredheim OMS, Kaasa S, Fayers P, Saltnes T, Jordhøy M, Borchgrevink PC (2008). Chronic non-malignant pain patients report as poor health-related quality of life as palliative cancer patients. Acta Anaesthesiol Scand.

[30] Landmark T, Romundstad P, Dale O, Borchgrevink PC, Vatten L, Kaasa S. Chronic pain: one year prevalence and associated characteristics (the HUNT pain study). Scand J Pain. 2013;4:182–7. 10.1016/j.sjpain.2013.07.022 29913652

[31] Price C, Lee J, Taylor AM, Baranowski AP (2014). Initial assessment and management of pain: a pathway for care developed by the British Pain Society. Br J Anaesth.

[32] Carnes D, Taylor SJC, Homer K, Eldridge S, Bremner S, Pincus T (2013). Effectiveness and cost-effectiveness of a novel, group self-management course for adults with chronic musculoskeletal pain: study protocol for a multicenter, randomized controlled trial (COPERS). BMJ Open.

[33] Craig P, Dieppe P, Macintyre S, Michie S, Nazareth I, Petticrew M (2008). Developing and evaluating complex interventions: the new Medical Research Council guidance. BMJ.

[34] Craig P, Dieppe P, Macintyre S, Michie S, Nazareth I, Petticrew M. Developing and evaluating complex interventions: new guidance. https://www.mrc.ac.uk/documents/pdf/complex-interventions-guidance/. Accessed 15 Mar 2016.

[35] Chan AW, Tetzloff JM, Altmann DG, Dickersin K, Moher D (2013). SPIRIT 2013: new guidance for content of clinical trial protocols. Lancet.

[36] Merskey H, Bogduk N (2004). Classification of chronic pain: descriptions of chronic pain syndromes and definitions of pain terms.

[37] International Association for the Study of Pain, Subcommittee on Taxonomy (1986). 2. Classification of chronic pain. Descriptions of chronic pain syndromes and definitions of pain terms. Pain Suppl.

[38] Bower P, Kennedy A, Reeves D, Rogers A, Blakeman T, Chew-Graham C (2012). A cluster randomised controlled trial of the clinical and cost-effectiveness of a ‘whole systems’ model of self-management support for the management of long-term conditions in primary care: trial protocol. Implement Sci.

[39] Turk DC, Gatchel RJ (2002). Psychological approaches to pain management.

[40] Thorn B (2004). Cognitive therapy for chronic pain. A step-by-step guide.

[41] McCracken L, Eccleston C (2003). Coping and acceptance. What to do with chronic pain?. Pain.

[42] Dragesund T, Råheim M (2008). Norwegian psychomotor physiotherapy and patients with chronic pain: patients’ perspective on body awareness. Physiother Theory Pract.

[43] Polit DF, Beck CT (2012). Nursing research. Generating and assessing evidence for nursing practice.

[44] Schulz K, Altman DG, Moher D (2010). CONSORT 2010 Statement: updated guidelines for reporting parallel group randomized trials. BMC Med.

[45] Galdas P, Darwin Z, Kidd L, Blickem C, McPherson K, Hunt K (2014). The accessibility and acceptability of self-management support interventions for men with long term conditions: a systematic review and meta-synthesis of qualitative studies. BMC Public Health.

[46] WMA. WMA Declaration of Helsinki – Ethical principles for medical research involving human subjects. 1964. http://www.wma.net/en/30publications/10policies/b3/index.html.pdf?print-media-type&footer-right=[page]/[toPage]. Accessed 15 Mar 16.

[47] The Norwegian Directorate of Health. ICPC-2. The Internationale classification for primary care. 2014. http://www.kith.no/sokeverktoy/icpc-2/bok/index.html. Accessed 16 Mar 2016.

[48] Hibbard JH, Mahoney ER, Stockard J, Tusler M (2005). Development and testing of a short form of the Patient Activation Measure (PAM). Health Serv Res.

[49] Insignia Health. Patient activation measure. Oregon; 2015.

[50] Hibbard JH, Mahoney ER, Stock R, Tusler M (2007). Do increases in patient activation result in improved self-management behaviors?. Health Serv Res.

[51] Hibbard J, Gilburt H (2014). Supporting people to manage their health. An introduction to patient activation.

[52] Eikelenboom N, Lieshout J, Wensing M, Smeele I, Jacobs AE (2013). Implementation of personalized self-management support using the self-management screening questionnaire SeMaS; a study protocol for a cluster randomized trial. Trials.

[53] Rijken M, Heijmans M, Jansen D, Rademakers J (2014). Development in patient activation of people with chronic illness and the impact of changes in self-reported health: results of a nationwide longitudinal study in the Netherlands. Patient Educ Couns.

[54] Grønning K, Skomsvoll JF, Rannestad T, Steinsbekk A (2012). The effect of an educational programme consisting of group and individual arthritis education for patients with polyarthritis – a randomised controlled trial. Patient Educ Couns.

[55] Steinsbekk A (2008). Måling av effekt av pasientopplæring. Norwegian version of Patient Activation Measure (PAM). Tidsskr Nor Laegeforen.

[56] Cleeland CS, Osoba D (1991). Pain Assessment in cancer. Effect of cancer on quality of life.

[57] Dworkin RH, Turk DC, Wyrwich KW, Beaton D, Cleeland CS, Farrar JT (2008). Interpreting the clinical importance of treatment outcomes in chronic pain clinical trials: IMMPACT recommendations. J Pain.

[58] Keller S, Bann CM, Dodd SL, Schein J, Mendoza TR, Cleeland CS (2004). Validity of the Brief Pain Inventory for use in documenting the outcomes of patients with noncancer pain. Clin J Pain.

[59] Tan G, Jensen MP, Thornby JI, Shanti BF (2004). Validation of the Brief Pain Inventory for chronic nonmalignant pain. J Pain.

[60] Klepstad P, Loge JH, Borchgrevink PC, Mendoza TR, Cleeland CS, Kaasa S (2002). The Norwegian Brief Pain Inventory questionnaire: translation and validation in cancer pain patients. J Pain Symptom Manage.

[61] Hølen JC, Lydersen S, Klepstad P, Loge JH, Kaasa S (2008). The Brief Pain Inventory: pain’s interference with functions is different in cancer patients compared with noncancer chronic pain. Clin J Pain.

[62] Kapstad H, Rokne B, Stavem K (2010). Psychometric properties of the Brief Pain Inventory among patients with osteoarthritis undergoing total hip replacement surgery. Health Qual Life Outcomes.

[63] McCormack HM, Horne DJL, Sheather S (1988). Clinical applications of visual analogue scales: a critical review. Psychol Med.

[64] Nicholas MK (2007). The Pain Self-Efficacy Questionnaire: taking pain into account. Eur J Pain.

[65] Nicholas MK, Asghari A, Corbett M, Smeets RJEM, Wood BM, Overton S (2012). Is adherence to pain self-management strategies associated with improved pain, depression and disability in those with disabling chronic pain?. Eur J Pain.

[66] Meisingset I, Woodhouse A, Stensdotter AK, Stavdahl Ø, Lorås H, Gismervik S (2015). Evidence for a general stiffening motor control pattern in neck pain: a cross sectional study. BMC Musculoskelet Disord.

[67] Nicholas MK, Asghari A, Blyth FM (2008). What do the numbers mean? Normative data in chronic pain measures. Pain.

[68] Zigmond AS, Snaith RP (1983). The hospital anxiety and depression scale. Acta Psychiatr Scand.

[69] Myhr A, Augestad LB (2013). Chronic pain patients – effect on mental health and pain after a 57-week multidisciplinary rehabilitation program. Pain Manag Nurs.

[70] Mykletun A, Stordal E, Dahl AA (2001). Hospital Anxiety and Depression (HAD) scale: factor structure, item analyses and internal consistency in a large population. Br J Psychiatry.

[71] Pallant JF, Bailey CM (2005). Assessment of the structure of the Hospital Anxiety and Depression Scale in musculoskeletal patients. Health Qual Life Outcomes.

[72] The EuroQoL Group (1990). EuroQoL – a new facility for the measurement of health-related quality of life. Health Policy.

[73] Herdman M, Gudex C, Lloyd A, Janssen MF, Kind P, Parkin D (2011). Development and preliminary testing of a new five-level version of EQ-5D (EQ-5D-5L). Qual Life Res.

[74] Obradovic M, Lal A, Liedgens H (2013). Validity and responsiveness of EuroQoL-5 dimension (EQ-5D) versus Short Form-6 dimension (SF-6D) questionnaire in chronic pain. Health Qual Life Outcomes.

[75] Schaller A, Dejonghe L, Haastert B, Froboese I (2015). Physical activity and health-related quality of life in chronic low back pain patients: a cross-sectional study. BMC Musculoskelet Disord.

[76] Solberg TK, Olsen JA, Ingebrigtsen T, Hofoss D, Nygaard ØP (2005). Health-related quality of life assessment by the EuroQoL-5D can provide cost-utility data in the field of low-back surgery. Eur Spine J.

[77] Bell IR, Cunningham V, Caspi O, Meek P, Ferro L (2004). Development and validation of a new global well-being outcomes rating scale for integrative medicine research. BMC Complement Altern Med.

[78] Eriksson M, Lindström B (2005). Validity of Antonovsky’s sense of coherence scale: a systematic review. J Epidemiol Community Health.

[79] Rikli RE, Jones CJ (2013). Development and validation of criterion-referenced clinically relevant fitness standards for maintaining physical independence in later years. Gerontologist.

[80] Tveter AT, Dagfinrud H, Moseng T, Holm I (2014). Health-related physical fitness measures: reference values and reference equations for use in clinical practice. Arch Phys Med Rehabil.

[81] Skou ST, Simonsen ME, Odgaard A, Roos EM (2014). Predictors of long-term effect from education and exercise in patients with knee and hip pain. Dan Med J.

[82] Becofsky K, Baruth M, Wilcox S. Physical functioning, perceived disability, and depressive symptoms in adults with arthritis. Arthritis. 2013. doi:10.1155/2013/525761. 10.1155/2013/525761 PMC3777208 24093063

[83] Rademakers J, Nijman J, Hoek L, Heijmans M, Rijken M (2012). Measuring patient activation in the Netherlands: translation and validation of the American short form Patient Activation Measure (PAM13). BMC Public Health.

[84] Maindal HT, Sokolwski I, Vedsted P (2009). Translation, adaption and validation of the American short form Patient Activation Measure (PAM13) in a Danish version. BMC Public Health.

[85] Zill JM, Dwinger S, Kriston L, Rohenkohl A, Härter M, Dirmaier J (2013). Psychometric evaluation of the German version of the patient activation measure (PAM13). BMC Public Health.

[86] Nossum R, Rise MB, Steinsbekk S (2013). Patient education – which parts of the content predict impact on coping skills?. Scand J Public Health.

[87] Solomon M, Wagner SL, Goes J (2012). Effects of a web-based intervention for adults with chronic conditions on patient activation: online randomized controlled trial. J Med Internet Res.

[88] Greene J, Hibbard JH (2012). Why does patient activation matter? An examination of the relationships between patient activation and health-related outcomes. J Gen Intern Med.

[89] Turner A, Anderson JK, Wallace LM, Bourne C (2015). An evaluation of a self-management program for patients with long-term conditions. Patient Educ Couns.

[90] StataCorp (2015). Stata Statistical software: Release 14.

[91] IBM Corp. IBM SPSS Statistics for Windows, version 23.0. Armonk; 2015.

[92] R Core Team (2016). R: A language and environment for statistical computing.

[93] Malterud K (2012). Systematic text condensation: a strategy for qualitative analyses. Scand J Public Health.

[94] Packer TL, Boldy D, Ghahari S, Melling L, Parsons R, Osborne RH (2012). Self-management programs conducted within a practice setting: who participates, who benefits and what can be learned?. Patient Educ Couns.

[95] Nolte S, Elsworth GR, Newman S, Osborne RH (2013). Measurements issues in the evaluation of chronic disease self-management programs. Qual Life Res.

[96] Turk DC, Dworkin RH, Revicki D, Harding G, Burke LB, Cella D (2008). Identifying important outcome domains for chronic pain clinical trials: an IMMPACT survey of people with pain. Pain.

[97] Nolte S, Osborne RH (2013). A systematic review of outcomes of chronic disease self-management interventions. Qual Life Res.

[98] Lewin S, Glenton C, Oxman AD (2009). Use of qualitative methods alongside randomised controlled trials of complex health-care interventions: methodological study. BMJ.

