# Supplementary material for: Expectations, effect and experiences of an easily accessible self-management intervention for people with chronic pain: study protocol for a randomised controlled trial with embedded qualitative study
Source: Trials. 2016 Jul 18;17:325. doi: 10.1186/s13063-016-1462-6 (PMC4950079; doi:10.1186/s13063-016-1462-6)
Supplement: Additional file 1: — Guidelines for the self-management course. Description and support for instructors on how to deliver the self-management course. (DOCX 20 kb) [file 13063_2016_1462_MOESM1_ESM.docx]

## Guidelines for the self-management course

| DAY 1 12.30- 1500 | Materials/ methodology |  |
| --- | --- | --- |
| - Welcome - Introduction - Presentation of the participants and instructors   Lecture:   - What is pain?  Understand differences between acute and chronic pain - How does pain affect us? - Elements from CBT in relation to pain   BREAK  Lecture continuous:   - Aim for the course - My expectations - My everyday life   Task: Map your everyday activities in the everyday circle  Homework:  Fulfil your everyday circle  Movement exercises:   - Relaxation of the jaw region: Open wide- hold- release Making underbite Moving the jaw from side-to-side | Use slides and give handouts  Each gives a short presentation of themselves  Use slides- give handouts. Encourage to discussion and questions  Social interaction  Use slides- give handouts  Dialogue  Questions to ask:  What are your expectations to the course?  What do you want to achieve?  Ask if anyone wants to share their thoughts and experiences with the mapping | |

| DAY 2 12.30- 1500 | Materials/ methodology |
| --- | --- |
| - Welcome - Short repetition of day 1. - Go through homework assignment   Lecture:   - Today’s topic: My challenges - What stops me in achieving what I want? - My challenges: sleeplessness, pain,  others expectations, my own expectations,  fear of pain, time, economy?   Task: - Find solutions! Problem solving.  BREAK  Lecture continuous:   - My challenges- my solutions - The thoughts’ influence on everyday life - Elements from Cognitive behavioural therapy   Task:  Why is it important what I tell myself?  Movement exercises:   - Easing of tension: Relaxing using monotone and slow movements | Dialogue  Use slides- give handouts  Dialogue  Discussion Ask if anyone wants to share an experience  Social interaction  Use slides- give handouts  Dialogue  Ask if anyone wants to share thoughts and reflexions. |

| DAY 3 12.30- 1500 | Materials/ methodology | |
| --- | --- | --- |
| - Welcome - Short repetition of day 2.   Lecture:   - Today’s topic: How to better cope in everyday life? - What gives me energy? - How do I cope and manage challenges? - What choices do I have? - What encourage activation?   BREAK  Lecture continuous:   - Acceptance- self-efficacy- sortation - The “Should, ought-to and must-do’s” - How to say “no”. When would it be better to say “yes”? - Self-confidence; self-confidence, self-esteem  and self-image   Task:  My qualities and my skills.  Using elements from CBT:   - Which activities gives me better self-efficacy? - Which activities in my life gives and takes my energy?   Movement exercises:  - Easing of tension using stretch and release,   or hold and release. | Dialogue  Use slides- give handouts  Dialogue  Ask if anyone wants to share an experience  Social interaction  Use slides- give handouts  Dialogue  Ask if anyone wants to share thoughts and reflexions.  Reflections upon what happens with the breath and the tension when stretching, holding, realising and afterwards? |  |

| DAY 4 12.30- 1500 | Materials/ methodology |
| --- | --- |
| - Welcome - Short repetition of day 3.   Lecture:   - Today’s topic: Goal setting - What do I want to achieve? - What are my choices? - What motivates me in working towards my goals? - Why is this important for me? - What is a goal? SMART goals: specific, measurable, acceptable, realistic, time-scheduled   BREAK  Lecture continuous:   - How to make an action plan - Goals and subsidiary objectives   Task:  My action plan   - Set SMART goals for yourself. - Use subsidiary objectives as part of your action plan   Homework:  Fulfil your action plan.  Work on your goal setting.  Movement exercises:  - Different techniques for stretch and release  using one arm and one leg, using both arms and legs. | Dialogue  Use slides- give handouts  Repetition from day 2  Dialogue  Social interaction  Use slides- give handouts  Ask if anyone wants to share thoughts and reflexions on their action plan.  Reflections upon what happens with the breath and the tension when stretching, holding, realising and afterwards? |

| DAY 5 12.30- 1500 | Materials/ methodology |
| --- | --- |
| - Welcome - Short repetition of day 4. - Go through homework assignment   Lecture:   - Today’s topic: I can- I have a choice! - Repetition on day 1:  How to cope better in my everyday life? - How to make good choices: How can I manage challenges more appropriate? - How to talk over your own interpretations   BREAK  Lecture continuous:   - How to manage pain more appropriate - How do I think, interpret, and react? - What do I do about it? To talk over your  interpretations and thoughts. - Which strategies for management do I use?  Problem-oriented, emotion-oriented, avoidance.   Task:  How are you getting along with your goals?   - New goals? - Reconsider your action plan? - Reconsider your subsidiary objectives?   Movement exercises:   - Focus on contact with the foundation. From back- or a sitting position,  finding techniques for a good rest on the foundation. | Dialogue  Ask if anyone wants to share his or her goals and experiences on working with their action plan.  Use slides- give handouts  Dialogue  Discussion Ask if anyone wants to share an experience  Social interaction  Use slides- give handouts  Dialogue  Ask if anyone wants to share thoughts and reflexions.  Reflection upon what happens with the breathing and contact with foundation when using the different techniques? |

| DAY 6 12.30- 1500 | Materials/ methodology |
| --- | --- |
| - Welcome - Short repetition of day 5.   Lecture:   - Today’s topic: The way ahead - Summarize the whole course - The aim for the course: - understand pain - change focus from pain to functioning  - increased self-understanding, self-efficacy,   sense of coping and better self-image - get to know your own resources and   become active in managing your own health - increase activation level - Repetition of techniques and strategies we have taught, both theoretical and for movement exercises - Repetition on how to create smart goals and action plans   BREAK  Lecture continuous:   - The CBT elements we have used in the course (situation- interpretation-emotion->talk over) - How has the self-management course answered  to your expectations? - What is your standpoint now? - How will you use what you have learned? - Information on other activities at the HLC and in the  community | Dialogue  Use slides- give handouts  Dialogue and discussion      Social interaction  Use slides- give handouts  Dialogue  How has life been since day 1?  Ask if anyone wants to comment on the content and delivery of the course |

In addition, the movement exercises implies:

- Working on balance in a sitting, standing, and walking position.
- To rise from a sitting to a standing position using different techniques,
- Transferring weight from one side to the other, and from a backwards to a forward position.
- Bending and stretching knees and arms, rotation of upper part and lower part of the body.
- Massage with helping aids like sticks under the foot, balance-pad and balls with knobs.
